# Toward ubiquitous surgical assistance services via private 5G infrastructure

**DOI:** 10.1007/s11548-026-03749-2

**Published:** 2026-07-22

**Authors:** Philipp Schollmaier, Benjamin Hohlmann, Noah Wickel, Ha Uyen Nguyen, Okan Yilmaz, Alisa Kovler, Philipp Feodorovici, Ralf Wellens, Klaus Radermacher, Armin Janß

**Affiliations:** 1 Chair of Medical Engineering, RWTH Aachen University, Aachen, Germany; 2 SurgiTAIX AG, Herzogenrath, Germany; 3 Bonn Surgical Technology Center (BOSTER), University Hospital Bonn, Bonn, Germany; 4 Department of Thoracic Surgery, University Hospital Bonn, Bonn, Germany; 5 Ericsson GmbH, Herzogenrath, Germany

**Keywords:** Private 5G, Virtualization, Surgical assistance services, Edge cloud, AI, Thoracic surgery

## Abstract

**Purpose.:**

Advanced data processing methods, including *artificial intelligence* (*AI*), are increasingly integrated into clinical practice, improving treatment quality and reducing practitioner strain. These methods entail elevated computational demands, necessitating secure and efficient resource provision. This study investigates a wireless infrastructure based on *private 5G* networking capable of provisioning scalable computational resources, exemplified by *surgical assistance services* (*SAS*) requiring live feedback.

**Methods.:**

To this end, five exemplary *SAS* covering medical imaging, speech, and device data processing were deployed as virtualized applications on a *private 5G*-connected edge computing cluster. Latency and concurrent stream capacity were evaluated against a local processing baseline.

**Results.:**

The *private 5G* configuration supports up to five concurrent *4K* or 41 concurrent *720p* video streams with on-site-like responsiveness and guaranteed latencies. A *720p* end-to-end endoscopic *SAS*, covering local image capture, remote segmentation, and return, consistently achieves capture-to-display latencies below 290 ms.

**Conclusion.:**

The findings demonstrate how medical systems can wirelessly offload diverse computational workloads, satisfying responsiveness requirements for surgical applications despite a preliminary *private 5G* configuration. Compared to *Wi-Fi*, *private 5G* requires greater infrastructure effort but provides dedicated spectrum with reduced interference susceptibility, enhanced security, and temporal determinism, properties that may be of particular relevance for surgical navigation and robotic systems.

## Introduction

Advanced data processing routines, e.g., based on artificial intelligence (*AI*), have become increasingly relevant for modern clinical practice disciplines, improving treatment while reducing strain for healthcare practitioners [1, 2]. Exemplarily, in the surgical domain, *AI* applications have demonstrated added value throughout the entire clinical workflow [3]. Particularly, in *video* (*VAS*)- and *robot-assisted surgery* (*RAS*) advanced data processing methods have contributed to significant efficiency gains, reducing operative time by up to 25% and complication rates by about 30% [4].

The integration of such methods entails an increasing demand for computational resources, the provision of which can be categorized into three primary paradigms:

- **Standalone** systems (system-integrated processing) handle workloads on themselves
- **External data centers** or *external clouds* provide computational resources and associated services via the public internet
- **Edge servers** or *edge clouds* handle workloads close to the data sources (on-premise servers), leveraging private institutional networking infrastructures

While **system-integrated** processing provides simplicity, security, and lowest latencies, it may incur greater costs and maintenance effort and limits scalability and flexibility, especially regarding continuous upgrades to maintain state-of-the-art patient care. Non-shareable computing resources further reduce overall efficiency across distributed devices. Conversely, **external data centers** constitute a scalable and flexible paradigm facilitating seamless inter-institutional integration and compute capabilities through global providers. Local hardware dependencies are thereby reduced, system maintenance is simplified, and accessibility is enhanced [5]. Established cloud infrastructures typically scale in both capacity and performance, enabling flexible adaptation for future applications. But dependencies on third-party infrastructure, particularly networking and cloud hosting platforms, introduce inherent vulnerabilities and unpredictable risks. Moreover, the growing prevalence of proprietary ecosystems with limited interoperability and vendor lock-in tendencies further restricts institutional flexibility and contributes to rising long-term operational expenditures [6]. **Edge-cloud** conceptions combine immediacy and reliability of integrated processing with scalability and flexibility of infrastructures from external data centers, making them particularly suited for time-critical, data-intensive, and privacy-sensitive workloads [7]. Recent studies highlight improvements in workflow and efficiency while offering enhanced reliability and security, along with reduced response times in comparison with external data center implementations [8].

Effective low-latency offloading of clinical workloads critically depends on the underlying networking. While wired connections (e.g., fiber optic) suggest the highest performance, they limit mobility and can introduce safety risks. Coupled with emerging trends toward flexible and mobile setups, medical systems are increasingly driving interest in wireless connectivity [9].

Although wireless technologies, such as *Wi-Fi*, generally achieve high throughput, low latency, and energy efficiency, their dependence on *carrier sense multiple access with collision avoidance* (*CSMA*/*CA*) does not allow temporal determinism. Under realistic operating-room conditions with numerous coexisting devices, contention-based access cannot guarantee bounded latencies or interference isolation [10]. In contrast, *private 5G* offers controlled coverage and capacity, lower susceptibility to interference through dedicated spectra, strong security, and superior temporal predictability, representing a supplementary approach for applications where these properties are required [11].

*Public 5G* approaches in medicine are well studied, especially for telemedical applications. Despite demonstrated benefits of *private 5G* across various domains (e.g., smart manufacturing), robust empirical evidence for *SAS* via *private 5G* and virtualized edge-cloud computing remains scarce [12].

**Contribution.** This work examines the technical feasibility of flexibly providing computational capabilities exemplified with five *surgical assistance services* (*SAS*). The five *SAS* are deployed as virtualized applications within a *Kubernetes*-based edge server cluster leveraging *private 5G* networking. While comparison with external data center processing is omitted due to the aforementioned vulnerabilities, a comparison to the local, system-integrated processing paradigm is included.

**Table 1:** System and media pipeline configuration parameters

Parameter	Value
*Private 5 G network*	
Architecture	Standalone non-public network (SNPN)
Core	Ericsson Packet Core Gateway
Orchestration	Red Hat OpenShift (Kubernetes)
Indoor small cell (ISC)	Ericsson Radio Dot System (RDS)
Number of radio units	1
MIMO	4×4
Frequency spectrum	3.7-−3.8 GHz
Duplexing	TDD
UE modems	Quectel RM520N-GL (M.2)
Boxed PC	UP Squared 7000
Aggregation 5 G router	Ericsson Cradlepoint R1900
*Edge compute node (2 nodes)*	
Platform	HPE ProLiant DL380 Gen10+
CPU	Intel Xeon 5318Y
Memory	128 GB
GPU	2 × NVIDIA L40
*Local compute node*	
CPU	Intel Core Ultra 7 165 H
Memory	64 GB
GPU	NVIDIA RTX 5000 Ada Mobile (80 W)
*Media pipeline (experiments)*	
Video codec	H.264 (AVC)
Resolutions/frame rate	4K (3840×2160), 720p (1280×720) @ 30 Hz progressive
Color depth/chroma	8-bit, 4:2:0
Video transport	UDP
IP video en-/decoder	Teradek Prism Flex 4K MkII
Audio	RTP, 16-bit uncompressed, 44.1 kHz

## Materials and methods

### System overview

A *private 5G* network (*standalone non-public network*—*SNPN*) is established using a campus-central *Red Hat OpenShift*-based cluster that orchestrates components of a deployed *Packet Core Gateway*, which links to a demonstration operating room (*dOR*). The *dOR* operates, among others, a *5 G* indoor small cell that connects to *5 G User Equipment* (*UE*), i.e., medical devices. Two dedicated server nodes within the network infrastructure are exclusively designated for application workloads. A device trolley in the *dOR* accommodates several medical devices, such as high-frequency surgical equipment, endoscopic units, and displays. The device trolley is supplied with a *5 G* router that aggregates signals and transmits them to the edge application servers. The current implementation includes medical device data conforming to the *IEEE/ISO 11073* *Service-oriented Device Connectivity* (*SDC*) standard family, audio, and video signals. Further mobile *UE*s equipped with *5 G* modems are integrated for audio capturing. Setup details are provided in Table 1.

**Fig. 1:**
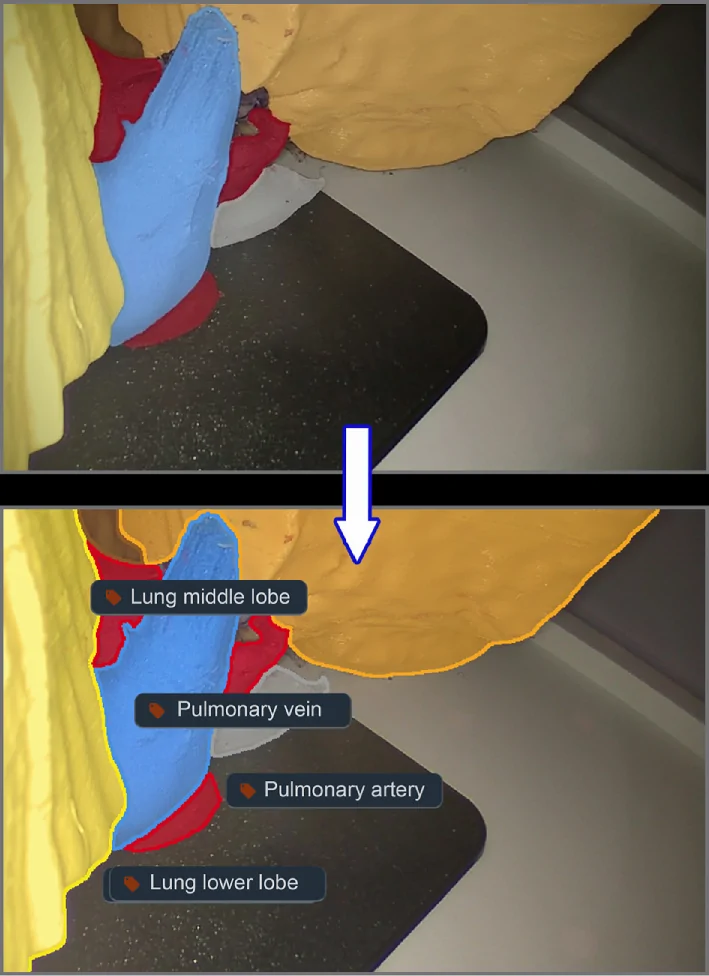
*Lung hilum anatomy instance segmentation service*

### Surgical assistance services

In particular, medical imaging live-feedback systems pose challenges to both the network infrastructure and subsequent data processing, as low-latency visual feedback is required. Therefore, *SAS*, particularly those involving medical imaging tasks, are developed. The *SAS* is virtualized within the edge server environment, accompanied by a dedicated transmission pipeline that enables low-latency interaction between distributed components.

Five *SAS* were developed, adapted, and deployed as exemplary workloads for the *5G edge-cloud* infrastructure. The selection deliberately spans from lightweight audio processing to computationally intensive multistage image analysis, to characterize platform versatility across diverse workload types.

- An ***in-body classification*** service, based on *SEResNet50* backbone (*MONAI* framework), enables regulatory-compliant broadcast and archiving of surgical video. This ensures that surgical video data can be broadcast or archived without violating data protection regulations. It constitutes a lightweight, standard image classification workload.
- A lung hilum anatomy ***instance segmentation*** service employs a *Mask2Former* architecture (*OpenMMLab*), providing orientational surgical support and representing a computationally intensive, state-of-the-art workload (Fig. 1).
- A cascading ***intersegmental plane*** (***ISP***) identification service combines monocular depth estimation for lung surface reconstruction with multistage non-rigid *ICP*-based registration of preoperative volumetric *CT* scans [13], overlaying *ISP*s onto thoracoscopic imaging to guide lung segment resection in *non-small cell lung cancer* (*NSCLC*) without specialized optics or fluorescent dyes. The multistage pipeline entails substantial computational demands.
- Two ***ultrasound segmentation*** services, employing *SwinUnet3D* and *DeepLabV3* architectures, delineate cortical bone structures from sonographic imaging across anatomical regions, guiding osteosynthesis screw and pin placement in trauma surgery [14].
- An audio processing service enables **voice control** for an endoscope, leveraging an *Emformer*-based approach, transforming spoken commands into *SDC*-compliant control instructions. The computational demands are rather lightweight.

### Data transmission pipeline

**Fig. 2:**
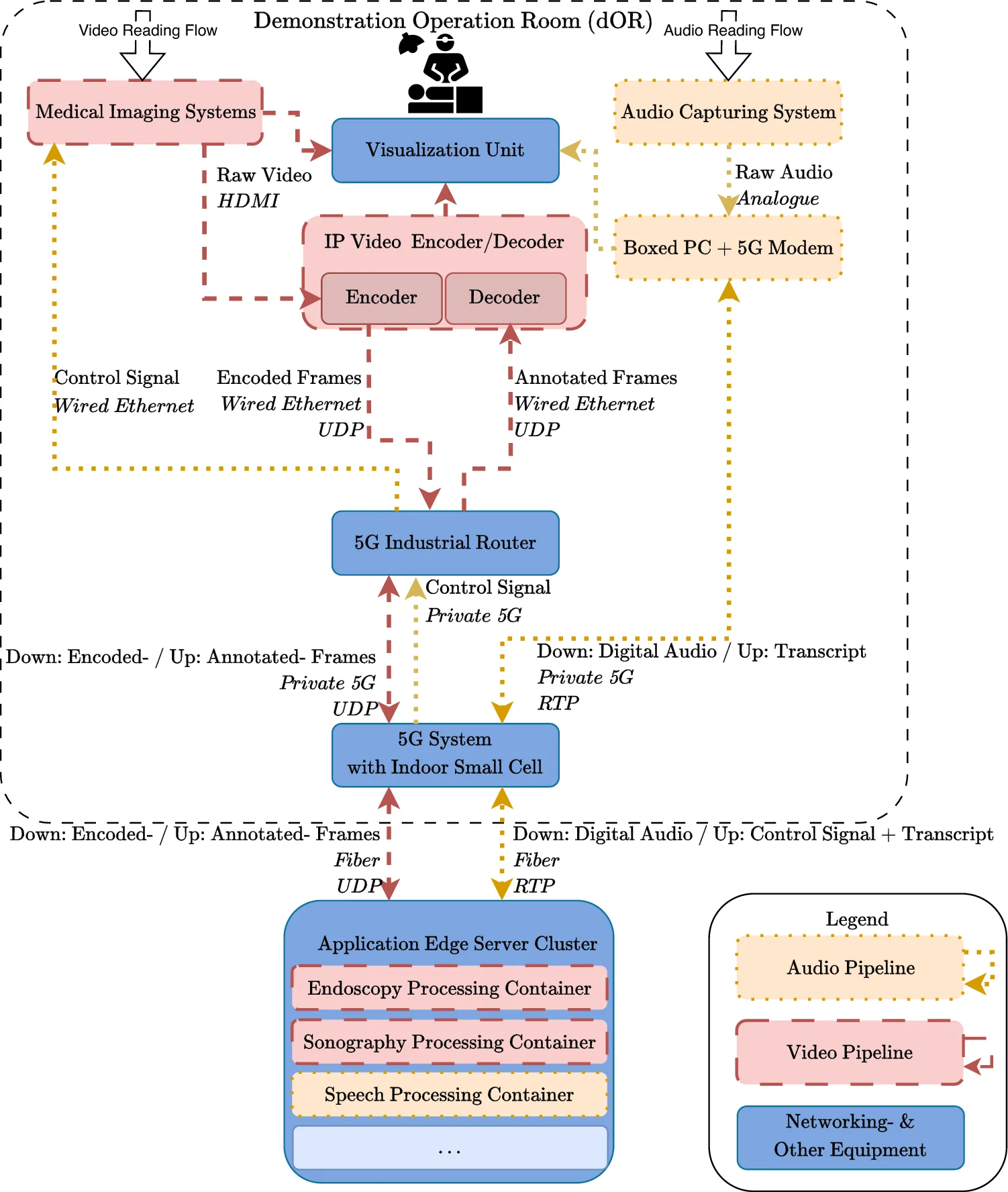
**Simplified System Conception**: (i.) Imaging (*red-dashed*, left) and (ii.) voice control (*yellow-dotted*, right) pipeline

For medical imaging, low-latency *IP* video en-/decoders capture, compress, and transmit video streams using *H.264* at resolutions up to *4K* (3840×2160) progressive scan 30 Hz, 8-bit color depth, and up to 4:2:2 chroma subsampling via *User Datagram Protocol* (*UDP*). Sonography inherently relies on single-channel grayscale imaging with spatial resolutions lower than *480p* at 15 Hz. For the audio pipeline, microphones capture voice commands and transmit them as chunk-wise packetized signals using *Real-time Transport Protocol* (*RTP*), employing uncompressed 16-bit audio at a sampling rate of 44.1 kHz. In the audio pipeline, the transcribed voice command is returned together with an inferred *SDC* control signal. Each *SAS* is virtualized for deployment on largely *commercial off-the-shelf* (*COTS*) hardware. Thereby, video encoding and decoding leverage the *GStreamer* and *FFmpeg* libraries, leveraging the *GPU* capabilities. The *SASs* receive *IP*-based video, audio, or medical device data for processing and subsequently return the results (i.e., labeled images or *SDC* commands). Processed image frames (e.g., segmentation masks) are provided within the same network via a lightweight *Flask*-based web service, while minimal transmission latency is achieved by directly returning frames to a designated *IP* video decoder unit in the *dOR*. The relevant details are listed in Table 1, and a simplified overview is illustrated in Fig. 2.

## Experiments and results

The experiments pursue two objectives: first, assessing the general technical feasibility and the perceived responsiveness of offloaded versus local or standalone processing across all surgical assistance services and second, characterizing the end-to-end *capture-to-display latency* (*CDL*) of the imaging pipeline and decomposing it into its frame transport and inference components. To contextualize the measured latencies against clinical requirements, maximum acceptable values of 320 to 400 ms have been reported for direct-feedback surgical applications, decreasing to approximately 100 ms in highly dynamic scenarios such as telemanipulative systems with direct control feedback [15–17].

**Feasibility and responsiveness**

Technical feasibility was demonstrated for all five *SAS*. A qualitative assessment involving two surgeons and three medical engineers evaluated the perceived responsiveness of local versus offloaded processing. None of the evaluators reliably distinguished between local and offloaded execution, indicating suitable responsiveness. For computationally intensive services, such as *ISP* segmentation, frame processing times extend to several seconds and constitute the primary bottleneck rather than the network offloading.

**Capture-to-display latency** (*CDL*)

**Fig. 3:**
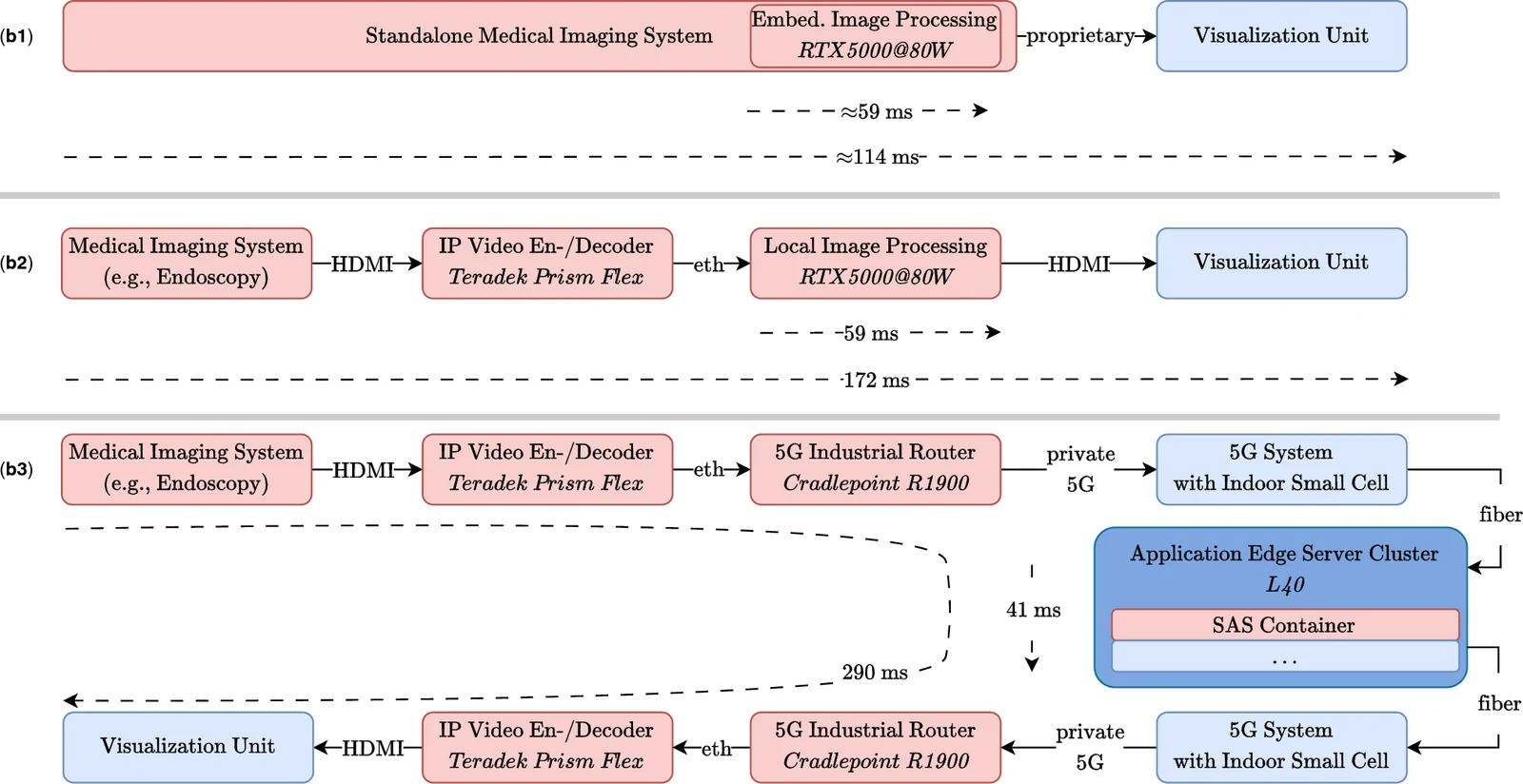
Imaging pipeline configurations (b1, b2, b3)

The *CDL* covers the complete data path from image capture to final display, comprising frame transport, including encoding, *IP* packetization, transmission, depacketization, decoding, and preprocessing steps such as color space conversion and array transformations, as well as the applied *SAS* inference operation. For clarity, these components are referred to as *transport* and *inference* throughout. Transport is measured for the imaging pipeline across three configurations of increasing scope, as depicted in Fig. 3, with *SAS* inference times measured separately. The inherent *CDL* (*glass-to-glass latency*) of standalone endoscopy systems without inference, reported at approximately 55 ms [17], serves as the transport reference for the *standalone* configuration (**b1**). Offloading frames via *IP* over a wired *Ethernet* connection (**b2**) or over the full *private 5 G* edge-cloud pipeline (**b3**) resulted in transport latencies of 113 ms and 249 ms, respectively, both excluding inference. The resulting total *CDL*s are summarized in Table 2 and illustrated in Fig. 3, also including inference times for the lung hilum segmentation *SAS*.

***SAS inference times***

Inference times vary substantially across services depending on their computational complexity. Segmentation is a common and computationally demanding task in medical imaging. Accordingly, the lung hilum instance segmentation *SAS*, representing a state-of-the-art workload, serves as the reference for inference characterization. Inference requires 41 ms on *AI* server-grade hardware (Table 1), applicable to the edge-cloud paradigm (**b3**), and 59 ms on expected per-*OR* hardware (Table 1), applicable to the standalone (**b1**) and local *IP* offloading (**b2**) setups. (Table 2, Fig.3)

**Table 2:** Capture-todisplay latencies for the three processing configurations (b1–b3)

Processing Paradigm	Transport	Inference	CDL
	(ms)	(ms)	(ms)
**(b1) System-integrated**	≈55	59	≈114
*RTX 5000 @ 80 W*			
**(b2) Local IP offloading**	113	59	172
*RTX 5000 @ 80 W*			
**(b3) Private 5 G edge server**	249	41	290
*NVIDIA L40*			

**Network capacity**

The current *private 5G* configuration theoretically sustains up to 41 concurrent *720p* (**c1**) or five concurrent *4K* (**c2**) video streams, reflecting the prospective scenario in which multiple operating rooms concurrently request surgical assistance services from the shared server nodes. As these figures reflect the currently employed *Time Division Duplex (TDD)* scheme, the *private 5 G* configuration could be further optimized to meet the specific requirements of individual hospitals.

## Conclusion and outlook

**Conclusion.** The proposed edge computing framework leveraging *private 5G* networking demonstrates a supplementary approach to meet growing computational demands for advanced data processing methods in clinical routines. The results further incentivize new modularization concepts for upcoming medical systems. *Private 5G* edge computing delivers secure, flexible, near on-site performance to all connected systems, merging data center adaptability with local immediacy. Despite the currently not fully performance optimized *private 5G* configuration, the current data and *SAS* pipeline achieve a stable *CDL* of 290 ms, which already satisfies latency requirements for direct-feedback applications but falls short for highly dynamic scenarios such as telemanipulative systems. The system’s modularization could furthermore reduce time-to-market, promote interoperability, and enhance accessibility, especially for small- and medium-sized enterprises (*SME*). Thereby, it fosters competition and accelerates clinical innovation by decoupling innovation and upgrade cycles of the system components. It should be noted that this work discusses technical aspects exclusively, without a detailed analysis of regulatory or certification hurdles.

**Outlook.** Besides forthcoming *5 G* configuration optimizations to reduce latency, shifting the current *Time Division Duplex* (*TDD*) scheme to an uplink-favored scheme seems favorable, as medical imaging and continuous physiological monitoring require predominantly uplink capacity. The current medical imaging transmission pipeline could particularly benefit by solely returning inferred labels incorporating client-sided frame-to-label association, which is more efficient than returning entire frames. Additional use case-specific preprocessing steps, such as cropping relevant frame areas, could further reduce processing times, network load, and overall computational demands. To leverage superior temporal predictability, a distinguishing feature of *5G* that sets it apart from other wireless technologies, future studies will incorporate applications such as surgical tracking and robotic systems that particularly require real-time guarantees.
